# Melatonin the "light of night" in human biology and adolescent idiopathic scoliosis

**DOI:** 10.1186/1748-7161-2-6

**Published:** 2007-04-04

**Authors:** Theodoros B Grivas, Olga D Savvidou

**Affiliations:** 1 Orthopaedic Department, "Thriasio" General Hospital, G. Gennimata Avenue, Magula, 19600 Greece

## Abstract

Melatonin "the light of night" is secreted from the pineal gland principally at night. The hormone is involved in sleep regulation, as well as in a number of other cyclical bodily activities and circadian rhythm in humans. Melatonin is exclusively involved in signalling the 'time of day' and 'time of year' (hence considered to help both clock and calendar functions) to all tissues and is thus considered to be the body's chronological pacemaker or 'Zeitgeber'.

The last decades melatonin has been used as a therapeutic chemical in a large spectrum of diseases, mainly in sleep disturbances and tumours and may play a role in the biologic regulation of mood, affective disorders, cardiovascular system, reproduction and aging. There are few papers regarding melatonin and its role in adolescent idiopathic scoliosis (AIS). Melatonin may play a role in the pathogenesis of scoliosis (neuroendocrine hypothesis) but at present, the data available cannot clearly support this hypothesis. Uncertainties and doubts still surround the role of melatonin in human physiology and pathophysiology and future research is needed.

## Background

### Pharmacology

**Figure 1:**
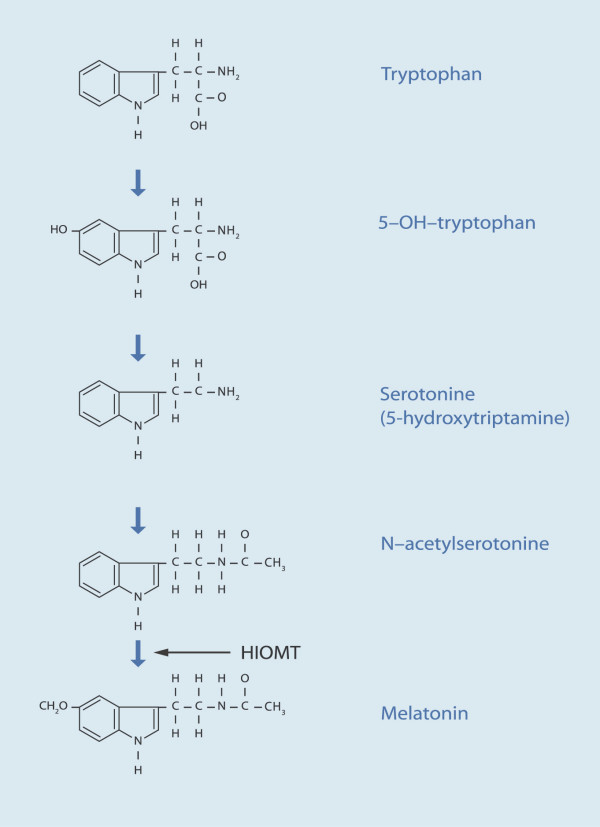
Biosynthesis of melatonin. Modified from [48].

**Figure 2:**
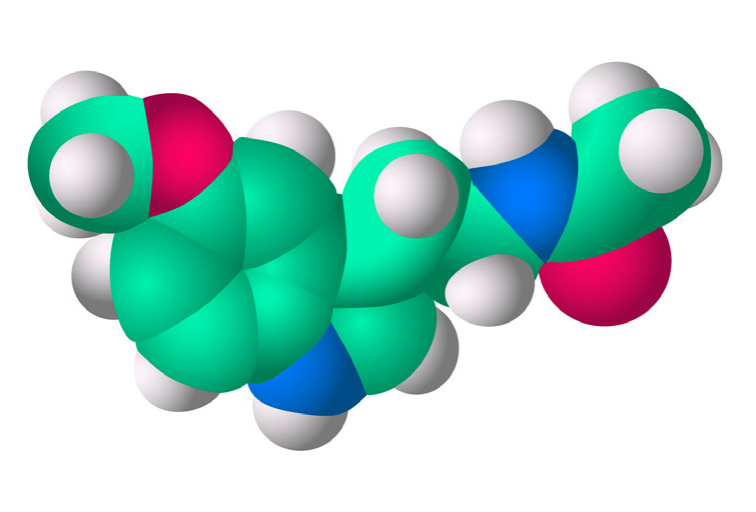
Melatonin molecule. (Modified from [86]).

The biological pathway of melatonin is not simple. In the biosynthesis of melatonin, or *N*-acetyl-5-methoxytryptamine, tryptophan is first converted by tryptophan hydroxylase to 5-hydroxytryptophan, which is decarboxylated to serotonin (figure 1 and 2). The synthesis of melatonin from serotonin is catalyzed by two enzymes (arylalkylamine *N*-acetyltransferase and hydroxyindole-*O*-methyltransferase) (HIOMT) that are largely confined to the pineal gland [1,2]. Melatonin is rapidly metabolized, chiefly in the liver, by hydroxylation to 6-hydroxymelatonin. The urinary excretion of 6-sulfatoxymelatonin (the chief metabolite of melatonin) closely parallels serum melatonin concentrations [3].

### Synthesis of melatonin-the role of light

In humans melatonin is produced mainly in the pineal gland and a small portion in the retina. The synthesis and release of melatonin are stimulated by darkness, melatonin is the "chemical expression of darkness" and inhibited by light [4]. Photic information from the retina is transmitted to the pineal gland through the suprachiasmatic nucleus of the hypothalamus (SCN) and the sympathetic nervous system [5]. During daylight hours the retinal photoreceptor cells are hyperpolarized and inhibit the release of norepinephrine [6]. With the onset of darkness the photoreceptors release norepinephrine, thereby activating the system, and a number of *a*1- and *b*1-adrenergic receptors in the gland increases [7]. The activity of arylalkylamine *N*-acetyltransferase, the enzyme that regulates the rate of melatonin synthesis, is increased, initiating the synthesis and release of melatonin. As the synthesis of melatonin increases, the hormone enters the bloodstream through passive diffusion.

Environmental lighting does not cause the rhythm but entrains it (alters its timing) [5]. Light has two effects on melatonin: day-night light cycles modify the rhythm of its secretion, and brief pulses of light of sufficient intensity and duration abruptly suppress its production [8]. In normal subjects, exposure to light inhibits melatonin secretion in a dose-dependent manner. The threshold is 200 to 400 lux (equivalent to ordinary fluorescent light), and maximal inhibition occurs after exposure to intense light (600 lux or higher) for one hour [9].

### Levels of melatonin

**Figure 3:**
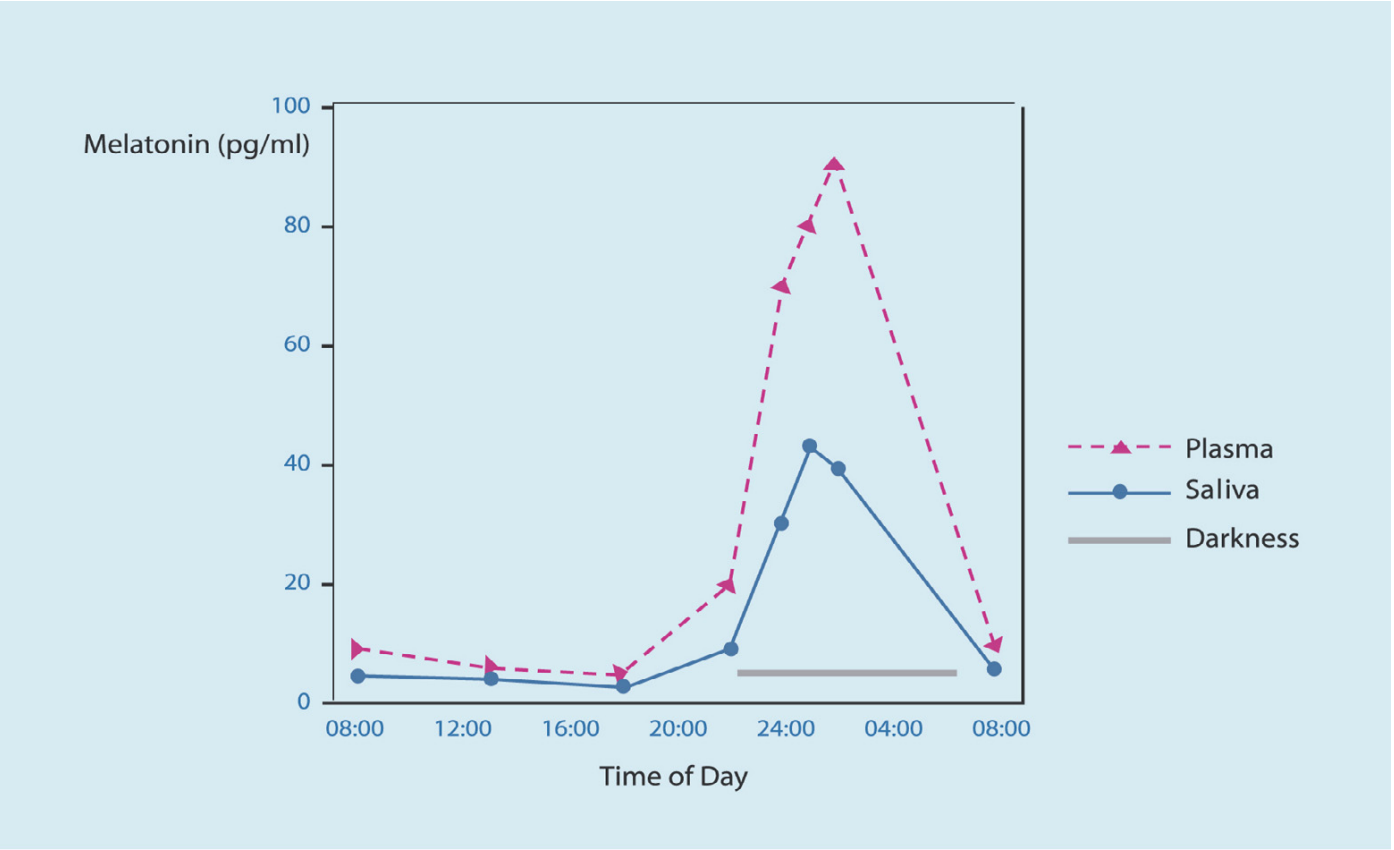
Diurnal variations of melatonin levels. The hormone secretion increases soon after the onset of darkness, peaks in the middle of the night, between 2 and 4 a.m., and gradually falls during the second half of the night. (Modified from [87]).

In humans melatonin has diurnal variations. The hormone secretion increases soon after the onset of darkness, peaks in the middle of the night, between 2 and 4 a.m., and gradually falls during the second half of the night (figure 3). This circadian rhythm of secretion plays an important role in its hormonal activity. In some endocrinopathies like Cushing disease this circadian rhythm of secretion of the hormone is absent. Several diseases such as: Klinefelter's syndrome, Turners syndrome, psoriasis vulgaris, myelomenigocoele, and sarcoidosis have been associated with disrupted melatonin profiles, both in terms of rhythm and magnitude [10].

#### Seasonal variations

There is a seasonal variation in human melatonin, with an earlier phase in summer and increased levels and duration of secretion in winter in high geographical latitudes [4].

#### Age variations

**Figure 4:**
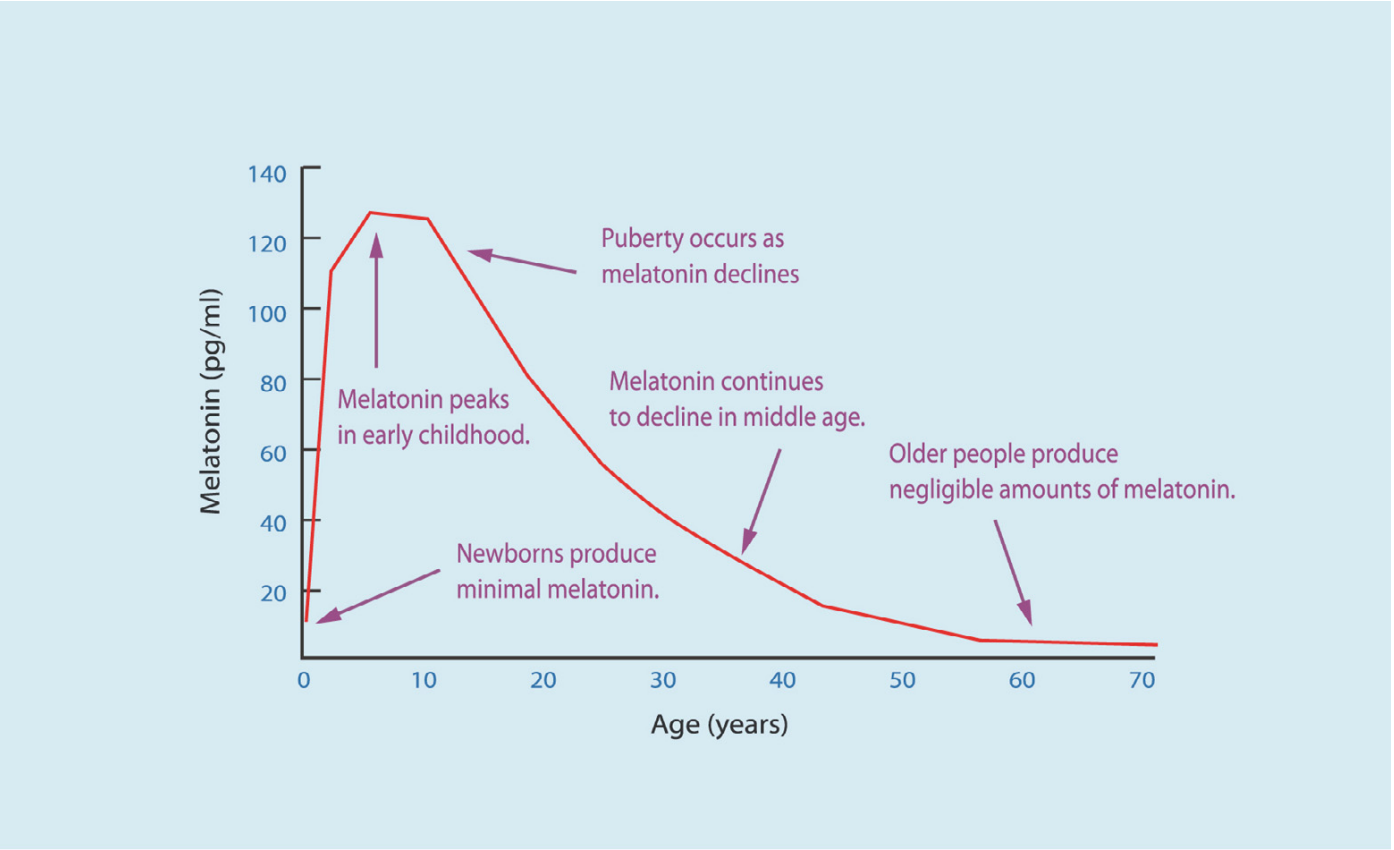
Age variations of melatonin levels. The hormone secretion increases in early childhood. In adolescent there is a decrease of the hormone concentration. The levels continued to decline gradually during middle age. In old population the levels of melatonin in serum are very low. (Modified from [88]).

Serum melatonin concentrations vary considerably according to age. Infants younger than three months of age secrete very little melatonin. Melatonin secretion increases and becomes circadian in older infants, and the peak nocturnal concentrations are highest (average, 325 pg per milliliter [1400 pmol per liter]) at the age of one to three years, after which they decline gradually 10–15% per decade [11] (figure 4). In normal young adults, the average daytime and peak night time values are 10 and 60 pg per millilitre (40 and 260 pmol per liter), respectively [3].

### The bioavailability of melatonin-sides effects

The bioavailability of orally administered melatonin varies widely. Oral doses (1 to 5 mg), which are now widely available in drugstores and food stores, result in serum melatonin concentrations that are 10 to 100 times higher than the usual night time peak within one hour after ingestion, followed by a decline to base-line values in four to eight hours. Very low oral doses (0.1 to 0.3 mg) given in the daytime result in peak serum concentrations that are within the normal night time range [12].

Melatonin is available in drugstores in tablets (0.2 mg, 0.3 mg, 0.5 mg, 1 mg, 2.5 mg, 3 mg, 5 mg), timed release tablets (1 mg, 2 mg, 3 mg) and liquid (1 mg/mL, 1 mg/4 ml). No serious side effects or risks (e.g., hypothermia, increased sleepiness, decreased alertness, and possibly reproductive effects) have been reported in association with the ingestion of melatonin. The dose-dependent physiologic effects of the hormone, however have not yet been properly evaluated in people who take large doses for prolonged periods of time. Despite the general absence of a marked endocrine action, decreased serum luteinizing-hormone concentrations and increased serum prolactin concentrations have been reported after the administration of pharmacologic doses of melatonin in normal subjects [13,14].

### Receptors of melatonin

The amphibian melatonin receptor was cloned in 1994, and cloning of the sheep and human receptors was reported shortly thereafter with high structural similarity (80%) between sheep and human clones [15,16]. Two membrane-bound melatonin-binding sites belonging to distinct groups have been identified: ML1 (high-affinity) and ML2 (low-affinity) receptors [17,18].

Melatonin receptors have been located in the suprachiasmatic nucleus, the pars tuberalis, and in the cerebellum [19,20]. Although the human cerebellum is not considered as an endocrine system, the external zone of the cerebellar molecular layer has the highest density of melatonin receptors: a convergency site for stimulatory and inhibitory afferents to the Purkinje cells. The Purkinje cells are the only output from the cerebellar cortex and are inhibitory for vestibular, oculomotor, and cerebellar neurons [19,21].

Activation of ML1 melatonin receptors, which belong to the family of guanosine triphosphate-binding proteins, a family that includes the serotonin and b-adrenergic receptors, results in the inhibition of adenylate cyclase activity in target cells [22,23]. ML1 receptors are of clear importance to the nervous system. The greatest density of high-affinity melatonin receptors (ML1) in humans are located in the suprachiasmatic nucleus of the hypothalamus (SCN). These receptors are responsible for chronobiological effects at the SCN, the circadian pacemaker. Within SCN, melatonin reduces neuronal activity in a time -dependant manner.

**Figure 5:**
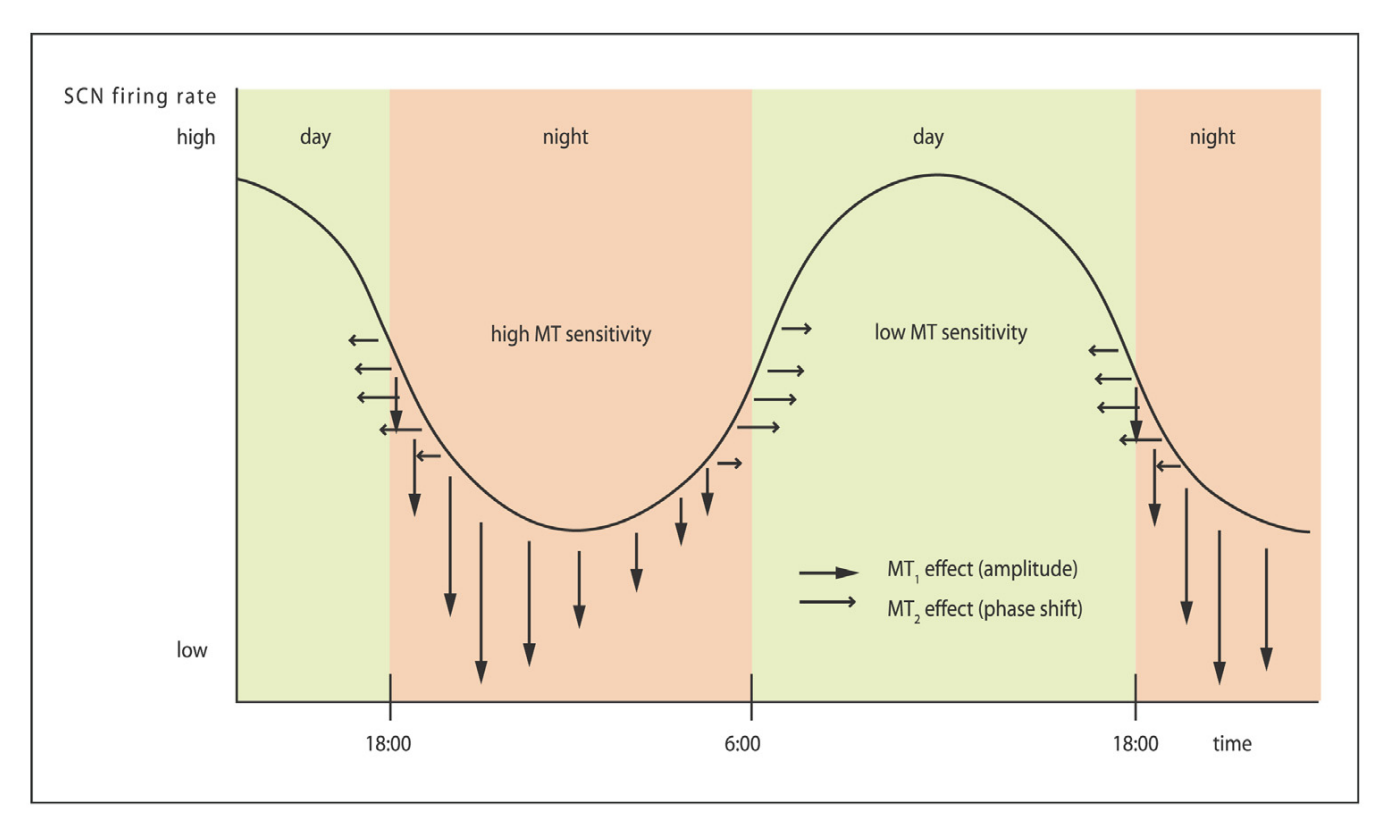
Effects of exogenous melatonin on nucleus suprachiasmatic neuronal activity (SCN). The effects of melatonin on SCN activity are mediated by at least two different receptors. They are insensitive during the day, but sensitive at dusk and dawn. MT2 causes phase shifts and during early night period MT1 decrease neuronal firing rate and amplitude. (Modified from [89]).

**Figure 6:**
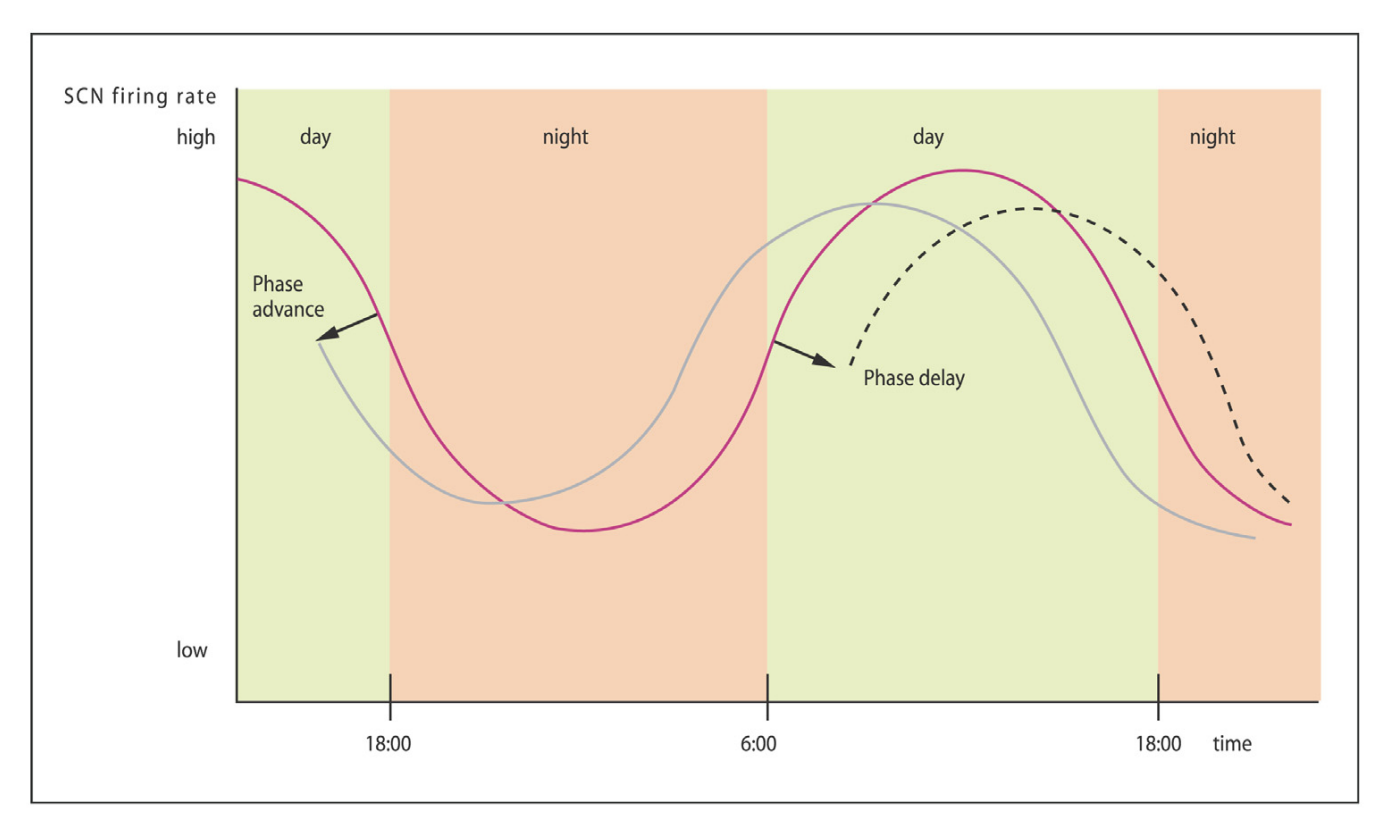
Effects of exogenous melatonin on nucleus suprachiasmatic neuronal activity (SCN). At the beginning of the night melatonin causes a phase advance (dashed line) and in the early morning hours causes a phase delay (dotted line)-MT2 receptors. (Modified from [89]).

**Figure 7:**
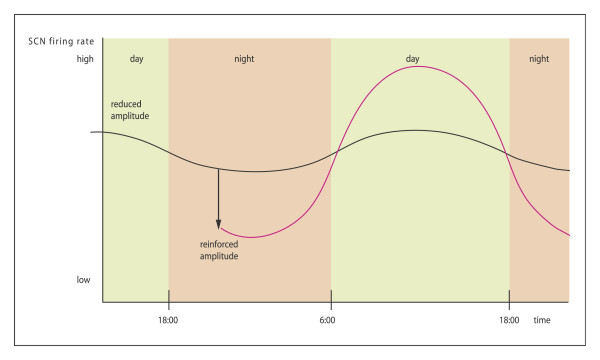
Effects of exogenous melatonin on nucleus suprachiasmatic neuronal activity (SCN). Melatonin administered during the early night period may resynchronize SCN neurons and thereby reinforce amplitude of SCN output signal without an effect on circadian phase-MT1 receptors. (Modified from [89]).

With the use of the polymerase chain reaction (PCR), two subtypes of a high-affinity melatonin receptor (ML1), were cloned from several mammals, including humans: the Mel1a and Mel1b receptors, which are now referred to as the MT1 and MT2 receptors, respectively [15,24]. This opens large new perspectives and approaches not only for the study of the mechanism of action of melatonin but also for the development of new molecules for therapeutic use. Several major actions of melatonin are mediated by the membrane receptors MT1 and MT2. They belong to the superfamily of G-protein coupled receptors containing the typical seven transmembrane domains. MT1 receptors activate protein kinase C-b, whereas MT2 receptors inhibit the soluble guanylate cyclase pathway while stimulating protein kinase C. MT1 receptors have the most widespread distribution in the rodent brain, account for the majority of melatonin-binding sites in most target tissues, and are widely believed to account for many melatonin actions in the brain. The MT2 receptor does not appear to be necessary for photoperiodic responses in hamsters [24]. These receptors are members of a new receptor group that is distinct from other G-protein-linked groups. The effects of melatonin on SCN activity are mediated by at least two different receptors. They are insensitive during the day, but sensitive at dusk and dawn. MT2 causes phase shifts and during early night period MT1 decrease neuronal firing rate and amplitude [25] (figure 5, 6, 7).

The ML2 receptors are coupled to the stimulation of phosphoinositide hydrolysis, are a distinct molecular species, but their distribution has not been determined. The ML2 (also named MT3) receptor was poorly characterized and enigmatic until a recent study identified it as a form of quinone reductase. This enzyme is widely distributed in different tissues and across different species. The importance of melatonin binding to this enzyme is unclear. Specific melatonin agonists are becoming available, which lack binding to the MT3/quinone reductase receptor; these agents will help to isolate the specific effects mediated by the high-affinity melatonin receptors, and may become important options for pharmacologic treatment of insomnia without the potential for side effects from interactions with the MT3/quinone reductase binding site [23].

**Figure 8:**
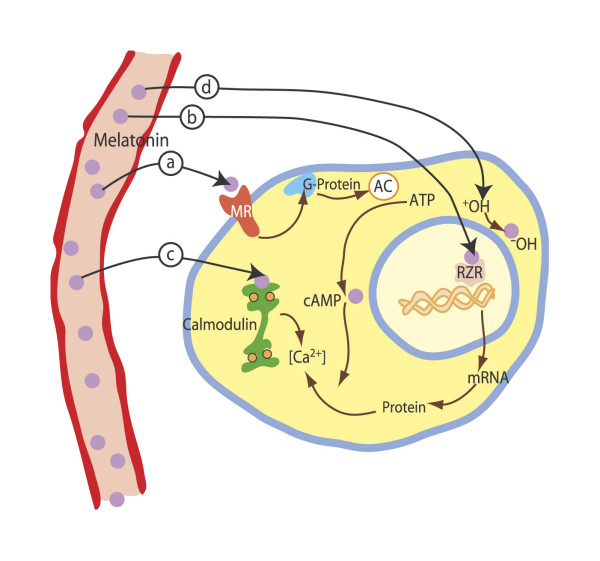
Through binding to calmodulin, melatonin may directly affect calcium signalling.(Modified from [90]).

Melatonin may also act at intracellular sites. Through binding to calmodulin (figure 8) the hormone may directly affect calcium signalling by interacting with target enzymes such as adenylate cyclase and phosphodiesterase, as well as with structural proteins [26]. There are melatonin receptors in various regions of the human brain and in the gut, ovaries and blood vessels [27-29]. Non-neural melatonin receptors (such as those located in the pars tuberalis of the pituitary) probably regulate reproductive function, especially in seasonally breeding species. Receptors located in peripheral tissues (e.g., arteries) may be involved in the regulation of cardiovascular function and body temperature. MT1 seems to mediate mainly vasoconstriction, whereas MT2 mainly causes vasodilation.

Moreau at al [30] investigated the melatonin signal transduction pathway and demonstrated that melatonin signalling is clearly impaired in osteoblasts of all patients with idiopathic scoliosis.

## Role of melatonin in human biology

### Circadian rhythm, jet lag and night shift

In humans the circadian rhythm for the release of melatonin from the pineal gland is closely synchronized with the habitual hours of sleep. Alterations in synchronization due to phase shifts (resulting from airline flights across time zones or unusual working hours) are correlated with sleep disturbances.

A phase shift in endogenous melatonin secretion occurs in airplane passengers after flights across time zones [31], in night-shift workers [32], and in patients with the delayed-sleep-phase syndrome (delayed onset of sleep and late waking up) [33].

The ingestion of melatonin may alter the normal circadian rhythm of melatonin secretion, but the reports on this effect are inconsistent, probably because of variations in the timing of the administration of melatonin in relation to the light-dark cycle [5]. The administration of melatonin in the early evening results in an earlier increase in endogenous night time secretion [34]. Exogenous melatonin appears to have some beneficial effects on the symptoms of jet lag, although the optimal dose and timing of ingestion have yet to be determined [5].

### Sleep disturbances

Serum melatonin concentrations were found to be significantly lower, with later peak night time concentrations, in elderly subjects with insomnia than in age-matched controls without insomnia [35].

Ingestion of melatonin affects sleep propensity (the speed of falling asleep), as well as the duration and quality of sleep, and has hypnotic effects. Oral administration of five mg of melatonin caused a significant increase in sleep propensity and the duration of rapid-eye-movement (REM) sleep [36,37]. In other studies, sleep propensity was increased in normal subjects given much lower doses of melatonin (0.1, 0.3, or 1 mg), and sleepiness in the morning was not increased [12]. The time to the maximal hypnotic effect varies linearly from about three hours at noon to one hour at nine p.m. [38]. The administration of melatonin for three weeks in the form of sustained-release tablets (1 mg or 2 mg per day) may improve the quality and duration of sleep in elderly persons with insomnia [39]. The hypnotic effect of melatonin may thus be independent of its synchronizing influence on the circadian rhythm and may be mediated by a lowering of the core body temperature [40].

Ophthalmic exams show that elevated intraocular pressure and large cup-to-disk ratios (index of optic nerve damage in patients with glaucoma) are independently associated with earlier melatonin timing (early phase). Lower illumination exposure also has independent associations with earlier melatonin timing. Conceivably, ophthalmic and illumination factors might have an additive effect on the timing of melatonin excretion, which in turn might predispose individuals to experience early morning awakenings. It is evident that daily illumination, ophthalmic factors, sleep duration, and race each have independent associations with melatonin. The observation that Blacks have lower illumination exposure, greater ophthalmic dysfunction, and higher 6-sulfatoxymelatonin levels merits further empirical study [41].

### Affective disorders

Although humans are no photoperiodic, bright light is essential for the suppression of melatonin and the phase shift of the circadian rhythm [42]. Low night time serum melatonin concentrations have been reported in patients with depression [43] and patients with seasonal affective disorder have phase-delayed melatonin secretion [44]. Although bright-light therapy (BLT) reduced the depression scores of such patients in one study, a direct association with the phase-shifting effect of light on melatonin secretion was not substantiated [42].

### Sexual maturation

Abnormally high or pharmacologic concentrations of melatonin in women are associated with altered ovarian function and anovulation. It is believed that the hormone also has antigonadal or antiovulatory effects in humans, as it does in some seasonal and nonseasonal mammalian breeders. Melatonin acts in gonades indirectly, reducing the secretion of gonadotropines and mainly LH [45]. The menarche is related with episodic secretion of LH during the night [46,47]. Melatonin may play a role in the timing of puberty and the onset of puberty in humans may be related to the decline in melatonin secretion that occurs as children grow [5].

The age at menarche in healthy population regressed by northern latitude showed that there is a statistical significant correlation (p < 0,001). For lower latitudes (as for example in Australia) there are no, at least recent, data available for the prevalence of scoliosis. Late age at menarche is noted in northern geographic latitudes and in Inuits (Eskimos) and the age of menarche decreases as the latitude is approaching the 30°-25° then it increases again [48,49]. The amount of sunlight and the quality of light may play a major role for the different initiation of menses in above-mentioned latitudes. Probably the increased levels of melatonin in countries with poor light environmental conditions as in northern countries reduced the secretion of LH and causes delayed age at menarche. The elevated levels of melatonin may also explains the inhibition of ovulation in the Inuits (Eskimos) during the months of winter night period [48,49].

### Aging

The decrease in night time serum melatonin concentrations that occurs with aging, together with its multiple biologic effects, has led several investigators to suggest that melatonin has a role in aging and age-related diseases [50,51]. Studies in rats and mice suggest that diminished melatonin secretion may be associated with an acceleration of the aging process [52]. It is interesting to note that the majority of herbs used in traditional Chinese medicine for retarding age-related changes and for treating diseases associated with the generation of free radicals also contain the highest levels of melatonin [53]. However, the age-related reduction in night time melatonin secretion could well be a consequence of the aging process rather than its cause, and there are no data supporting an antiaging effect of melatonin in humans.

### Malignancy

There is evidence from experimental studies that melatonin influences the growth of spontaneous and induced tumours in animals. Pinealectomy enhances tumour growth, and the administration of melatonin reverses this effect or inhibits oncogenesis caused by carcinogens [54]. Melatonin in combination with interleukin 2 has been used in clinical trials in patients with malignant tumours [55]. Data on the relation between melatonin and oncogenesis in humans are conflicting, but the majority of the reports point toward protective action. Low serum melatonin concentrations and low urinary excretion of melatonin metabolites have been reported in women with estrogens-receptor-positive breast cancer and men with prostatic cancer [56-58]. The mechanism by which melatonin may inhibit tumour growth is not known.

### Cardiovascular system

The evidence obtained during the last years suggests that melatonin exerts certain effects upon the cardiovascular system. The presence of vascular melatoninergic receptors binding sites has been demonstrated. It has been shown that patients with coronary heart disease have a low melatonin production rate, especially those with higher risk of cardiac infarction and/or of sudden death. There are clinical data, reporting alterations of melatonin concentrations in serum in coronary heart disease. People with high levels of low-density lipoprotein (LDL)-cholesterol have low levels of melatonin. It has been shown that melatonin suppresses the formation of cholesterol, reduces LDL accumulation in serum and modifies fatty acid composition of rat plasma and liver lipids. People with hypertension demonstrate lower melatonin levels versus those with normal blood pressure. It is questionable whether the administration of the hormone will decline blood pressure to normal range [59].

### Bone protector

Melatonin may have a direct effect on bone. Suppression of melatonin secretion lowered serum calcium concentration, an effect prevented by melatonin administration. Treatment of ovariectomized rats with melatonin prevents bone loss by an effect partly dependent on residual estradiol levels. Melatonin presumably acts as an autacoid in bone cells since it is present in high quantities in bone marrow, where bone cell precursors are located. Melatonin dose-dependently augments proteins that are incorporated into the bone matrix, like procollagen type I c-peptide. Osteoprotegerin, an osteoblastic protein that inhibits the differentiation of osteoclasts is also augmented by melatonin in vitro. Melatonin through its free radical scavenger and antioxidant properties may impair osteoclast activity [60].

### Interaction with Calmodulin

Many investigators have noted abnormalities in the structure and the function of thrombocytes in patients with idiopathic scoliosis. Those with larger scoliotic curves have a higher concentration of a more dense type of platelet, compared with those with small curves or control subjects [61]. Calmodulin is a calcium-binding receptor protein, and it regulates the contractile properties of muscles and platelets. Increased calmodulin levels in platelets have been shown to be associated with the worsening of AIS. Melatonin binds to calmodulin and has been shown to act as a calmodulin antagonist. The relationship seems to be complex. Nikaido, et al [62] showed that several calmodulin antagonists inhibited nocturnal melatonin synthesis in chick pineal cells. Their results supported the hypothesis that melatonin acts as a calmodulin antagonist and that cellular functions may be rhythmically regulated by melatonin modulation of calmodulin dependent protein phosphorylation. Benitez-King et al [63] detected calmodulin immunoreactivity in ependymocytes in the follicular zone and in interstitial cells in the perifollicular zone in the chicken pineal. The lack of calmodulin immunoreactivity in pinealocytes raised questions about its proposed function in melatonin synthesis. Hazlerigg et al 1996 [64] supported the hypothesis that under physiological conditions, melatonin synchronizes different body rhythms through cytoskeletal rearrangements mediated by its calmodulin antagonism.

## Melatonin and adolescent idiopathic scoliosis

The cause of adolescent idiopathic scoliosis (AIS) in humans remains obscure and probably multifactorial. Át present there is no proven method or test available to identify children or adolescent at risk of developing AIS or identify which of the affected individuals are at risk of progression. Reported associations are linked in pathogenesis rather than etiologic factors. A number of suggestions concerning its aetiology have been proposed including neuromuscular, connective tissue structure, vestibular dysfunction, melatonin secretion, platelet microstructure, mechanical, growth related and developmental, asymmetry in the brainstem, genetic factor, equilibrium dysfunction and impairment of proprioception leading to the idea that a disturbance of postural control but no single factor has been identified so far. Many authors think that a relation exists between the origin of scoliosis and balance troubles. Visual impairment has been shown to increase the prevalence of idiopathic scoliosis in human subjects when compared to the prevalence of the general population [65-70]. Research is needed to better define the role of all factors in AIS development.

Melatonin may play a role in the pathogenesis of scoliosis (neuroendocrine hypothesis) but at present, the data available cannot clearly show the role of melatonin in producing scoliosis in humans.

Machida et al found that pinealized chickens developed scoliosis and attributed this effect to decreased melatonin production. They show that scoliosis could be prevented by administration of melatonin. [71,72]. In another study they found significantly decreased levels of melatonin (actual serum levels with multiple regular overnight samples) in five adolescents with progressive curves, while those with stable curves were similar to controls [73]. They postulate that melatonin bonded calmodulin, thereby affecting calcium metabolism with muscle cells and thereby causing asymmetry in paraspinal muscle growth and development. The findings in pinealectomised chickens introduced the 'melatonin-deficiency hypothesis' as a possible cause of human AIS. Later Hilibrand et al [74] using morning and evening urine samples assayed for melatonin were unable to confirm differences between adolescent females with idiopathic scoliosis and controls. Contrary to their hypothesis, the urinary melatonin was actually slightly higher in the scoliosis patients than in the controls. The four patients in their study who subsequently had progressive curves did have lower values than the patients with stable curves or the controls, however the difference was not statistically significant (p < 0.25). Bagnall et al [75] also measured melatonin levels in adolescents with idiopathic scoliosis. In a small group (7 patients, 7 controls) of older patients with scoliosis, he was unable to show statistically significant differences in melatonin levels with 2 am and 2 pm serum samples. The authors remarked that their patients were 2–3 years beyond their point of maximum progression of curvature and that studies of adolescents during the maximal growth phase would be important. They suggested that melatonin's activity may be through the intermediation of growth hormone. Fagan et al [76] measured the 24 hour urinary melatonin production in patients with AIS. No significant difference in diurnal, nocturnal or total urine 6-sulphatoxy melatonin excretion was found between adolescent patients with a stable spinal deformity (AIS) or severe deformity requiring surgery and controls of similar age and gender. The author suggests that absolute levels of melatonin in an individual are less likely to be important than the rhythm of secretion. Brodner et al[77] measured the levels of serum melatonin and the excretion of urinary 6-hydroxy-melatonin-sulphate, the principal metabolite of melatonin in nine patients with adolescent idiopathic scoliosis and in ten age-and gender-matched controls. There were no statistically significant differences in the secretion of serum melatonin or the excretion of urinary 6-hydroxy-melatonin-sulphate between the patients and the control group. Cheung et al [78] reports that none of the 18 pinealectomised monkeys developed scoliosis in a mean follow up period of 28 months, and it strongly suggests that the possible etiologic factors producing idiopathic scoliosis in lower animals are different from primates, and that findings in lower animals cannot necessarily be extrapolated to human beings. Using the indirect way by examining the metabolite of melatonin, the urinary 6-sulfatoxyl-melatonin, as well as the pineal gland metabolism, where the melatonin is produced, by the assessment of the glucose metabolism using F-18 FDG brain PET(F-18 fluorodeoxyglucose (FDG) brain positron emission tomography (PET)) it was shown that there was no significant difference in melatonin secretion and no melatonin deficiency between the patients with AIS and the control group [79].

Thus the data regarding human melatonin levels is mixed at best and the melatonin deficiency as a causative factor in the aetiology of scoliosis cannot be supported. The biological relevance of melatonin in AIS is controversial because: a) no significant decrease in circulating melatonin level has been observed in a majority of studies, b) experimental pinealectomy did not lead systematically to a scoliosis in all pinealectomised chicken, c) melatonin injections in pinealectomised animals did not always prevent the formation of scoliosis.

There are good physiologic reasons why the chicken model cannot be simply extrapolated to the human situation. The distribution of melatonin receptors in the chicken is more widespread than in other species. They are found throughout the brainstem and in the dorsal grey matter of the signal cord, particularly in the lumbar region. Receptors have even been demonstrated in the chicken ovary and testicle. In humans no extrapineal source of melatonin production has been shown to affect the circadian rhythm of hormone levels. In humans pinealectomy leads to a loss of the night time melatonin peak and a drop in basal levels below detection, whereas in chickens, it only eliminates the night time peak. Although melatonin serves as the zeitgeber for many animals, its actions appear to differ between humans, other mammals, and other vertebrates [74,76]. Moreover idiopathic scoliosis patients do not have documented immune deficiency or difficulties with sleep, two deficiencies, which might be expected if melatonin was decreased. Looking at the accessible to us peer review literature there was no report of any sleep disturbances in children with AIS. Children who were operated for scoliosis don't show increase incidence of postoperative infections compare with the incidence of other operations in the same age group of people, something that someone would expect if these children have immunodeficiency due to lower levels of melatonin. Murphy et al [80] studied children who were hospitalized for the management of idiopathic scoliosis (IS) and neuromuscular scoliosis (NMS) via analysis of the 2000 Healthcare Cost and Utilization Project Kid Inpatient Database. Children with NMS more frequently developed pneumonia (3.5% vs. 0.7%, p < 0.001), respiratory failure (24.1% vs. 9.2%, p < 0.001), urinary tract infections (5.3% vs. 0.7%, p < 0.001), and surgical wound infections (1.3% vs. 0.3%, p < 0.001). Coe et al [81] in a retrospective study used Scoliosis Research Society (SRS) morbidity and mortality data submitted for surgical cases operated from 2003 to 2005 to determine whether scoliosis subtype (degenerative vs. idiopathic) is an independent predictor of complications and mortality in surgery for adult scoliosis by an analysis of the SRS database of complications as submitted by its members. The study demonstrated that the complication rate for surgery for degenerative scoliosis is significantly higher than idiopathic scoliosis (15,6% vs. 12,3% respectively). The difference was statistically significant (p < 0.01). In another study of 2876 patients who were treated surgically for spinal deformity the infection rate was 2,4%, while degenerative scoliosis had an infection rate of 6,2%, neuromuscular scoliosis had 3,6%, adult idiopathic scoliosis had 1,6%, while adolescent idiopathic scoliosis (AIS) had an infection rate of only 0,9% [82].

Another implication for the melatonin deficiency hypothesis is that increase incidence of scoliosis has not been observed in children after pinealectomy or pineal irradiation because of pineal neoplasias, although they have luck of serum melatonin [83,84]. Murata et al [84] analyzed secretion of melatonin and pituitary hormones in 14 patients with germinoma originating in the pineal or the hypothalamic-neurohypophyseal region. Germinoma cells originating from the pineal gland impair the production of melatonin by pineocytes and consequently induce a permanent melatonin deficiency in those patients, but these patients don't developed idiopathic scoliosis.

Moreau et al [30] performed in vitro assays with bone-forming cells isolated from 41 patients with AIS and 17 control patients exhibiting another type of scoliosis or non. Primary osteoblast cultures prepared from bone specimens obtained intraoperatively during spine surgeries were used to test the ability of melatonin and Gpp(NH)p, a GTP analogue, to block cAMP accumulation induced by forskolin. In parallel, melatonin receptor and Gi protein functions were evaluated. They found dysfunction in osteoblasts isolated from 100% of the patients with AIS tested. Classification of patients with AIS into 3 distinct groups suggested the presence of different mutations that could cause such dysfunction. Among possible causes, the authors suggest that abnormalities in Gi proteins function could be involved in AIS pathogenesis

The prevalence of scoliosis differs at different countries. In Northern latitude the prevalence is high compared with this towards 25–30° (Finland 9,2% -Greece 2,9%). The age at menarche regressed by latitude showed that there is a statistical significant correlation. The regression curves of prevalence of adolescent idiopathic scoliosis by latitude and age at menarche by latitude are of similar pattern [48].

The prevalence of scoliosis in a population of blind women was 42,3% while the prevalence in the general population in the same region is 2,9%. Melatonin may play a role in the pathogenesis of scoliosis [85]. It could be hypothesized that the levels of melatonin in this population (women in northern latitude, and blind women) causes delay sexual maturation and render the growing immature spine of these women to longer exposure, until maturity, to detrimental causative factors of scoliosis [85].

## Conclusion

Melatonin the "light of night" is not a simple hormone. It has many complex functions, which are only recently being defined. In comparison with other signalling molecules the numerous actions that have been attributed to melatonin are exceptional. Unfortunately there are differences in the pharmacology of melatonin between the species and different biological circadian rhythms. Chicken model cannot be simply extrapolated to humans. No permanent deficiency of secretion of melatonin occurs in patients with AIS. Evidence for a transient deficiency before and/or during development of scoliosis is scant and requires confirmation in a large number of subjects. The diurnal variations in melatonin levels and the circadian rhythm of secretion play an important role. There is now evidence that melatonin may have a role in biologic regulation of circadian rhythms, sleep, mood, and perhaps reproduction, tumour growth, cardiovascular system and aging. However, uncertainties and doubts still surround the role of melatonin. It will be an important issue of future research to investigate the role of melatonin in human biology, the clinical efficacy and safety of melatonin under different pathological situations.

## Abbreviations

**SRS**: Scoliosis Research Society

**FDG, PET**: F-18 fluorodeoxyglucose (FDG) brain positron emission tomography (PET)

**cAMP**: cyclic adenosine monophosphate

**Gpp (NH)p**: guanilyl 5'-imidophospate, nonhydrolysable analogue of GTP

**GTP**:guanosine 5'-triphosphate (cell and molecular biology) A nucleoside triphosphate that is instrumental in many cellular processes, including microtubule assembly, protein synthesis, and cell signalling, due to the energy it releases upon removal of its terminal phosphate group (producing guanosine 5'-diphosphate).

**Forskolin**: is a labdanediterpene that is produced by the plant plectranthus barbatus. Forskolin is commonly used to raise levels ofcAMP in the study and research of cell physiology. Forskolin resensitizes cell receptors by activating the enzyme adenylyl cyclase and increasing the intracellular levels of cyclic Adenosine Monophosphate (cyclic AMP or cAMP). Cyclic AMP is an important signal carrier that is necessary for the proper biological response of cells to hormones and other extracellular signals. It is required for cell communication in the hypothalamus/pituitary gland axis and for the feedback control of hormones.

## Authors' contributions

TBG conducted the collection of literature, and involved in drafting the article. ODS conducted the collection of literature, and involved in drafting the article. Both of the authors read and approved the final manuscript.
